# Liver Transplantation for Nonresectable Colorectal Liver Metastases (CRLM)

**DOI:** 10.1007/s13193-023-01827-4

**Published:** 2023-10-16

**Authors:** Abdullah K. Malik, Balaji Mahendran, Rajiv Lochan, Steven A. White

**Affiliations:** 1 Institute of Transplantation, Freeman Hospital, Newcastle Upon Tyne Hospitals NHS Foundation Trust, Newcastle Upon Tyne, UK; 2 NIHR Blood and Transplant Research Unit, Newcastle University, Newcastle Upon Tyne, UK; 3 Department of Hepatobiliary and Liver Transplantation Surgery, Manipal Hospitals, Bangalore, India

**Keywords:** Liver transplantation, Colorectal cancer, Liver metastases, Oncology

## Abstract

Transplantation represents the most radical locoregional therapy through removal of the liver, associated vasculature and locoregional lymph nodes, and replacing it with an allograft. Recent evidence has demonstrated that transplantation for unresectable CRLM is feasible with acceptable post-transplant outcomes in a highly selected cohort of patients. Controversy exists regarding whether transplantation is an appropriate treatment for such patients, due to concerns regarding disease recurrence in the transplanted graft in an immunosuppressed recipient along with utilising a donor liver which are in short supply. Expanding the indications for liver transplantation may also limit access for other patients with end-stage liver disease having ethical implications due to the effect of increasing the waiting list. In this review, we summarise the current evidence for liver transplantation in patients with nonresectable CRLM and highlight unresolved controversies and future directions for this type of treatment.

## Introduction

Liver resection (and/or ablation) is the only curative-intent treatment for colorectal liver metastases (CRLM) [1–4]. Improvements in surgical technique, anaesthesia, oncology, and understanding of tumour biology have led to a significant evolution in the management of CRLM with 5-year survival following surgery ranging from 30 to 60%, depending on specific prognostic factors [2–7]. The definition of ‘resectable’ disease has significantly changed over the past 20 years with surgery becoming more extensive, facilitated through advancements in methods to grow the future liver remnant such as portal vein embolisation [8, 9], hepatic vein embolisation [10], and associating liver partition and portal vein ligation for staged hepatectomy [11] in addition to the RAPID procedure (resection and partial segment 2–3 liver transplantation with delayed total hepatectomy) [12], as well as using chemotherapy for conversion to operable. Despite all these revolutionary techniques up to 70% of patients with CRLM may still be considered unresectable [1, 13–15] based on tumour size, number of lesions, involvement/proximity to major vascular structures, and the size/function of the future liver remnant.

In contrast, liver transplantation for nonresectable CRLM represents the most radical form of locoregional therapy and may offer selected patients a potentially curative outcome. Generally most groups agree that prior to transplantation the primary must be resected with R0 margin confirmed on histopathology without any evidence of extra-hepatic disease [16–18]. The aims of patient selection for listing are to identify patients who are likely to benefit from liver transplantation and have favourable disease biology. Although increasing evidence has found transplantation to be feasible with acceptable outcomes in patients with liver limited disease [9, 19–22], controversy exists regarding the risk of disease recurrence, the impact on organ allocation to other patients on the waiting list, and the precise indications for listing patients with nonresectable CRLM.

In this review, we outline the current evidence-base regarding liver transplantation for nonresectable CRLM as well as discussing some of the current recommendations for listing patients for liver transplantation. We also highlight some of the challenges when adopting transplantation as an accepted treatment for nonresectable disease and the potential future landscape in transplant oncology in CRLM.

## Current Evidence

A large proportion of the available evidence comes from Norway [9, 19–21, 23, 24]. In the 1990s, the European Liver Transplant Registry provided early data on outcomes for patients with CRLM who underwent liver transplantation. The authors reported unacceptable 1- and 5-year survival rates of 62% and 18%, respectively [25–27], culminating in a moratorium on liver transplantation for CRLM.

In Norway, the number of deceased donor liver grafts exceeds the number of patients on the waiting list [24]. The surplus of donor liver grafts led to a re-exploration of expanding the indications for liver transplantation with respect to nonresectable CRLM, leading to the SECA trials. The SECA-1 trial was a prospective study of 21 patients who underwent liver transplantation for nonresectable CRLM [24], with no adjuvant chemotherapy administered post-transplantation. The main inclusion criteria were a complete primary resection, no evidence of extrahepatic disease, receipt of at least 6 weeks of chemotherapy, and ECOG performance status of 0 or 1. The authors reported 1- and 5-year overall survival rates of 95% and 60%, respectively, and concluded that liver transplantation was a feasible treatment option in some selected patients with nonresectable disease. A secondary analysis compared the 21 patients who underwent liver transplantation with a contemporary cohort who received palliative chemotherapy [19] and found that 5-year overall survival was significantly worse in the chemotherapy group (9%). Disease recurrence was observed in 19 out of the 21 patients during follow-up, 8 of whom developed pulmonary disease suitable for further curative intent treatment. The study identified pre-transplant maximum tumour size > 5.5 cm, pre-transplant carcinoembryonic antigen [28] level > 80 μg/L, progressive disease, and interval from primary resection to transplantation < 2 years as factors predictive of decreased survival. The Oslo score was developed based on these findings, with a higher score associated with worse outcome (each factor awarded 1 point, with maximum score of 4).

The SECA-2 trial [21] followed on from the SECA-1 pilot study, with stricter inclusion criteria (including maximum tumour size of 10 cm and at least 1 year from primary resection to transplantation) aimed at improving survival and recurrence rates. The trial included 15 patients with nonresectable CRLM, 4 of whom had previously undergone liver resection for CRLM. Overall survival at 1 and 5 years was reported as 100% and 83%, respectively. Eight patients developed recurrence with disease-free survival being 53% and 35% at 1 and 3 years, respectively. Six of the eight patients with recurrence developed pulmonary recurrence, of whom 5 underwent curative intent treatment. The authors further concluded that stricter selection criteria can be employed to improve outcome and that transplantation for nonresectable CRLM warrants further exploration.

A separate study examined the impact of expanding recipient and donor criteria [20], reporting a very short median disease-free and overall survival of 4 and 18 months, respectively. The Norwegian group also compared liver transplantation with portal vein embolisation and liver resection in patients with nonresectable CRLM [9]. The study compared 50 patients from the SECA trials with 53 patients who underwent portal vein embolisation and liver resection and subdivided groups into ‘low tumour load’ and ‘high tumour load’ (9 or more metastatic lesions or a maximum tumour diameter equal to or greater than 5.5 cm). Overall survival was significantly greater in patients undergoing liver transplantation compared to those subjected to portal vein embolization combined with liver resection in the high tumour load group (median overall survival 40.5 months vs. 19.2 months). The results of this study may suggest that the true benefit of transplanting patients with nonresectable CRLM is in those who have a high tumour burden.

The SECA trials possess several limitations. Firstly, there is likely to be a high risk of selection bias given the lack of randomisation and small numbers. Secondly, following liver resection for CRLM, the standard of care is adjuvant chemotherapy [2, 13, 29]. In the SECA trials, adjuvant chemotherapy post-transplantation was not administered, which may not reflect current practice as adjuvant chemotherapy has been shown to improve disease-free survival following resection. Finally, Norway uniquely has a surplus of donor livers available; therefore, expanding current indications for transplantation is less likely to have an impact on other patients on the waiting list compared to most other countries where there is a shortage of donor livers relative to the waiting list. This may limit application of this protocol in other countries.

The impact of pre-transplant chemotherapy and/or locoregional therapies raises interesting issues regarding the biology of disease. In the SECA-2 trial, potential recipients were required to have received first-line chemotherapy with a specific tumour size and appropriate response requirements. Arguably, this will inherently have led to the group selecting patients with biologically favourable disease who may have experienced extended survival on chemotherapy had they not undergone transplantation. The majority of patients experienced disease recurrence in the lungs, with an indolent course further demonstrating a biologically favourable phenotype. Overall survival following relapse exceeded 5 years in the majority of cases [30].

Transplantation for nonresectable CRLM could be considered a palliative treatment given that recurrence is almost inevitable [31]; however, the longer overall survival following transplantation in comparison to patients treated with chemotherapy alone could justify this approach; however, further prospective data is awaited. Additionally, as chemotherapy alone is associated with much shorter overall survival compared to transplantation [1, 13, 14, 19] (despite potential confounding by selection bias in the reported studies), there may not be clinical equipoise between the two treatments which would make a trial randomising participants to transplantation or chemotherapy potentially unethical. The TRANSMET trial (NCT02597348) is an ongoing multi-centre French trial randomising patients to receive chemotherapy followed by liver transplantation or chemotherapy alone. The results of such a trial will be very important in determining whether liver transplantation is superior to chemotherapy alone. Similarly, the SECA-3 trial (NCT03494946) in Norway will randomise patients to liver transplantation or chemotherapy, transarterial chemoembolization, selective internal radiation therapy, and/or other available treatments, further expanding on the Norwegian experience.

Where Norway is unique in having a surplus of donor liver grafts, other countries are likely to experience longer waiting times for a deceased donor liver graft. A North American group explored the use of living donor liver transplantation for treating patients with nonresectable CRLM [22]. The study prospectively followed up 10 cases from 3 North American institutions and reported a 1.5-year overall survival of 100% and disease-free survival of 62%. The study enrolled patients with low Oslo and clinical risk scores, selecting cases with highly favourable tumour biology to reduce recurrence risk. The use of live donors aims to reduce pressures on the waiting list and logistically allows transplantation to be scheduled following completion of chemotherapy, whereas the availability of deceased donor liver grafts leads to a degree of unpredictability as to when transplantation will occur. However, the use of live donors as a source of liver grafts for patients with nonresectable CRLM is controversial due to the relatively high risk of disease recurrence and potential risk to the donor. Caution is warranted in interpreting the results of this trial due to the high risk of selection bias and limitations in extrapolating the data to external cohorts, given the small number of patients studied. However, the results of this trial are promising and may open a new avenue of treatment for patients with nonresectable CRLM.

More recently, Sasaki et al. reported a series of 46 patients who underwent transplantation for unresectable CRLM in 5 centres in North America [32], with half undergoing living donor liver transplantation. This retrospective study based on UNOS data reported favourable outcomes, with 1- and 3-year overall survival 89.0% and 60.4%, respectively. Disease-free survival was reported as 75.1% and 53.7% at 1 and 3 years, respectively. Although the results of this study are promising, limitations include a high risk of selection bias given the small number of patients and limited follow-up time. There was limited information available from the UNOS database on pre-transplant loco-regional or systemic therapies administered, which may have also confounded the results.

## Indications for Listing

Currently, there are no universally accepted criteria for listing patients with nonresectable disease for liver transplantation. Transplant-related survival benefit can be defined as the difference between mortality risk following transplantation and mortality risk without transplantation. Listing strategies aim to enable access to transplantation in those patients who are most likely to benefit. As waiting list pressures significantly vary between countries, the aims of transplantation and acceptable post-transplant outcome will differ. Countries with longer waiting times and low number of deceased donors will likely employ stricter criteria compared to countries with shorter waiting times and a greater number of available donors including access to LDLT programs [31]. The implication of too strict listing criteria is limiting access to transplantation in patients who may have some benefit. Alternatively too lax criteria may result in futile transplants taking place, potentially wasting precious deceased donor liver grafts. Living donation may allow for criteria to be relaxed to widen access in some countries; however, the risk of donor morbidity and mortality must be carefully considered and balanced with the risk of futility.

The Norwegian group looked at 3 scoring systems (Oslo Score, Fong Clinical Risk Score, and total PET liver uptake) to identify which scores predicted better long-term outcome in patients with nonresectable disease [30]. The analysis identified low scores in the 3 systems (Oslo Score 0–2, Fong Clinical Risk Score 0–2, and low total PET liver uptake) as predictive of improved survival, with the Fong Clinical Risk Score being the most restrictive and limiting access to liver transplantation to 30% of the entire cohort.

Although internationally individual listing criteria is likely to vary between countries, the International HPB Association (IHPBA) recognised the need for consensus guidelines and published recommendations regarding listing criteria, sequence of treatments, graft selection and post-transplant immunosuppression [17]. Briefly, the consensus guidelines recommend that all patients with nonresectable CRLM (discussed by a specialist hepatobiliary multidisciplinary team) should have had complete R0 resection of the primary tumour, local recurrence excluded with colonoscopy within 3 months of transplantation, exclusion of extrahepatic disease through cross-sectional imaging, observed response to first-line chemotherapy for at least 6 months, and absence of disease progression during bridging therapy. Particular emphasis is placed on observation of potential recipients during bridging therapy, to ensure disease biology will not result in futility. The guidelines recommend against re-transplantation for recurrent CRLM in patients following liver transplantation, with the Norwegian group identifying recurrence within the transplanted liver with an adverse prognosis [33].

More recently, the UK Liver Advisory Group (part of NHS Blood and Transplant) established a fixed-term working unit to outline the criteria for liver transplantation in patients with nonresectable CRLM [16], aiming to transplant 20–30 patients over 2 years. The criteria were similar to those established by the SECA trials and the recommendations in the IHPBA consensus guidelines. Key differences are that patients listed for transplantation must demonstrate no evidence of disease progression for a minimum of 2 years following commencement of chemotherapy. Where the IHPBA guidelines recommend against listing patients with nonresectable disease that may be amenable to immunotherapy (deficiency of DNA mismatch repair genes and/or microsatellite instability), the UK guidelines make no such exclusion. Additionally, the IHPBA guidelines recommend against transplantation in patients with BRAF mutations, given the association with worse survival in metastatic colorectal cancer [34]. The UK guidelines do not exclude potential recipients with BRAF mutations. This is likely due to the other stringent criteria applied in ensuring no evidence of disease progression in the 2-year preceding transplantation selecting potential recipients with nonaggressive disease although in most cases BRAF mutation patients usually display aggressive disease [34, 35].

## Selection of Liver Grafts to Patients with Nonresectable CRLM

Expanding the indications for liver transplantation will reduce the relative number of grafts available for other patients on the waiting listing countries that do not have a surplus of deceased donor liver grafts. This has implications for waiting time and mortality risk for the entire waiting list population. Patients with nonresectable CRLM are likely to have preserved liver function and low MELD scores and may not be prioritised in a ‘sickest first approach’. Prolonged waiting time to transplantation may result in significant disease progression leading to de-listing. Living donation [22] and the use of expanded criteria donors may provide additional grafts. In particular normothermic machine perfusion allows for high-risk liver grafts to undergo viability assessment [36] and may improve utilisation of liver grafts considered to be ‘high-risk’. The VITTAL trial demonstrated that following viability assessment, up to 70% of liver grafts deemed unsuitable for transplantation met viability criteria and were transplanted with 100% patient survival at 1 year [37]. The use of living donors may prove controversial given the high recurrence rate associated with transplantation. Futility must be avoided through appropriate selection of recipients as well as a thorough discussion regarding the surgical risks to both the donor and recipient, as well as recurrence risk. In the retrospective study by Sasaki et al., approximately 50% received live donor liver grafts, demonstrating that where allocation practices may not provide exception points or priority to patients with nonresectable CRLM, living donation may be used to ensure access to transplantation in suitable patients. Additionally, the deceased donor liver grafts transplanted were often from expanded criteria donors with a history of malignancy, steatosis, heavy drinking, or hepatitis with 1-year overall survival comparable to contemporary outcomes. A prospective trial from the Toronto group (NCT02864485) will explore outcomes in patients with nonresectable disease treated with neoadjuvant chemotherapy and live donor liver transplantation.

## Future Directions

Further international experience will aid decision-making regarding recipient and donor selection in liver transplantation for nonresectable CRLM. A prospective international registry is warranted to avoid centre-specific biases from influencing results. Future studies should evaluate what impact expanding the waiting list has on patients with other aetiologies. Changes to the allocation system are considered a ‘zero-sum’ game where providing an advantage to one group of patients inherently disadvantages another group. Regular evaluation and analysis of listing criteria and outcomes are necessary as more evidence and experience become available to ensure that futile transplants (wasting deceased donor liver grafts) are not taking place and patients potentially benefitting are not being denied access through too stringent listing criteria. Ongoing randomised and nonrandomised trials exploring liver transplantation for nonresectable CRLM will provide further evidence for this treatment and will likely demonstrate superiority over palliative chemotherapy.

## Declarations

### Dedication

This manuscript is dedicated to the memory of Professor Leslie H. Blumgart, in gratitude for his immeasurable contribution to the development of hepatobiliary and pancreatic surgery, and the training of countless surgeons.
